# Diminuendo! Tactics in Support of FaaS Migrations

**DOI:** 10.1007/978-3-030-58858-8_13

**Published:** 2020-08-18

**Authors:** Sebastian Werner, Jörn Kuhlenkamp, Frank Pallas, Niklas Anders, Nebi Mucaj, Olesia Tsaplina, Christian Schmidt, Kann Yildirim

**Affiliations:** 6 IT University of Copenhagen, Copenhagen, Denmark; 7 University of British Columbia, Vancouver, BC Canada; 8 Information Systems Engineering, Technische Universität Berlin, Berlin, Germany; 9 ProgPrak Team, Technische Universität Berlin, Berlin, Germany

**Keywords:** Serverless, Migration, FaaS, FaaSification

## Abstract

Function-as-a-Service (FaaS) receives close attention due to highly desirable characteristics, including pay-as-you-go pricing, high elasticity, and its fully managed nature. To leverage these benefits for existing applications, developers face the challenge of migrating legacy code to a FaaS platform (FaaSification). Unfortunately, however, actionable guidance on how to do so for real-world applications does not exist. In this paper, we report on our experience from FaaSifying a data-intensive application, and evaluating different options through extensive experimentation, using approaches such as regression tests and tracing. Based on the obtained results, we present five migration tactics in support of future FaaSification.

## Introduction

Function-as-a-Service (FaaS) is a new cloud execution model that receives close attention due to highly desirable characteristics, including pay-as-you-go pricing, millisecond elasticity, or provider-managed operational tasks for, e.g., deployment [6]. To leverage these benefits for existing applications, developers face the challenge of migrating legacy code to a FaaS platform (FaaSification).

While an increasing number of supported programming languages and relaxed limitations, e.g., maximum execution time, give the impression that FaaSification is a trivial task, first exploratory research [4, 7] indicates that leveraging the non-functional benefits of FaaS beyond correct execution requires a careful redesign, profiling, and configuration. Unfortunately, little information is available on how these and further subjects are (to be) addressed in the FaaSification of real-world web applications. Thus, many application developers are unaware of the different fallacies of FaaSification.

In this paper, we report on our experience from FaaSifying a data-intensive web application from a VM-based deployment. For the migration, we initially used a naïve migration approach presented in [7] that expectedly resulted in failing non-functional end-to-end regression tests. We were able to significantly reduce these degradations by 1) instrumenting application code for tracing purposes, 2) identifying the root-cause for degradation in different third-party libraries and some sections of legacy code, and, finally, 3) refactoring parts of the application architecture and the usage of third-party libraries.

To let others profit from our experiences, we synthesize our insights from the migration and additional previous research into five common migration tactics in support of future FaaSification: *Precompute*, *Reuse*, *Strip*, *Be Lazy*, and *Replace*.

## Application and Migration Goal

**Fig. 1.**
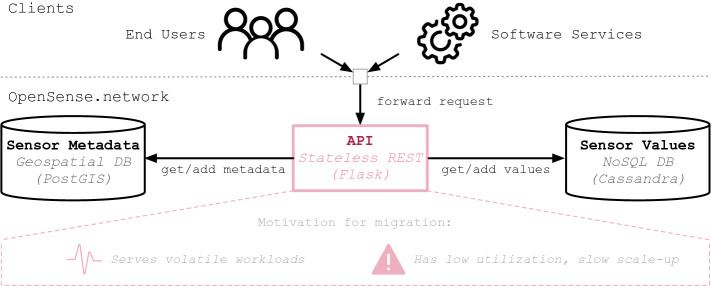
OpenSense.network architecture with selected component for migration.

We selected the open participatory data platform OpenSense.network [2] as a use-case to evaluate the effects of legacy code during the migration to FaaS environments. OpenSense.network offers a horizontally scalable, Flask-based API to let users contribute and access sensor data of globally distributed environmental sensors in a uniform, web-friendly way. To provide geospatial capabilities and high-volume sensor data in a performant and scalable manner, it employs a hybrid storage model, comprising a PostGIS database for static metadata and a Cassandra cluster holding timeseries of actual measurements (see Fig. 1). Both the API and databases of OpenSense.network are deployed on the TU Berlin data center premises. For the migration, we were particularly interested in moving the Flask API to a FaaS platform to free up computation resources when the API is not in use while at the same time being able to handle spiking loads in case of, for instance, occasional bulk inserts or data access surges. Further, we were interested in reducing operational overhead, e.g., the management of the Flask API virtual machines.

We selected Apache OpenWhisk^1^ as the target platform and deployed it in the same network as the current OpenSense.network deployment to allow connections to the same databases as the original APIs and, thus, to avoid the need for data migration.

To compare the behavior and responses of the migrated API to those of the original one, we additionally created and continuously extended a rich set of end-to-end regression tests.

## FaaS Migration Approach

In this section, we outline our approach in migrating OpenSense.network. We start by describing our initial naïve approach, followed by the steps taken to identify root-causes of performance degradations and the subsequent refactoring.

### Naïve Migration

In the first step of the migration, we followed a naïve reuse approach similar to Llyod et al. [7]. Accordingly, we implemented a custom runtime container, based on the OpenWhisk Python runtime^2^ with the necessary Python dependencies for OpenSense.network already built-in. These custom runtimes can reduce cold-start problems as less code needs to be downloaded and compiled initially.

Furthermore, we used a modified version of the *flask-openwhisk*^3^ wrapper to map OpenWhisk requests to Flask, resulting in a FaaS version of the pre-existing Flask API with almost zero modification.^4^ In line with our expectations and previous findings [7], this naïve FaaSification approach was successful on a functional level but exhibited significant performance degradation compared to the original deployment. For instance, a request for a single sensor value for the migrated API took between 2 and 20 s for cold and warm functions, respectively, while it took less than a second on the original API.

In the following, we first describe our approach to identifying the root-causes of this degradation.

### Regression Detection

Determining the root-cause of problems in FaaS applications is challenging [4] since FaaS platforms offer no out-of-the-box facilities for remote-debugging and -profiling. Instead, developers have to rely on application and system log information which can be limited in volume, making debugging and profiling tasks tedious and cumbersome.

**Fig. 2.**
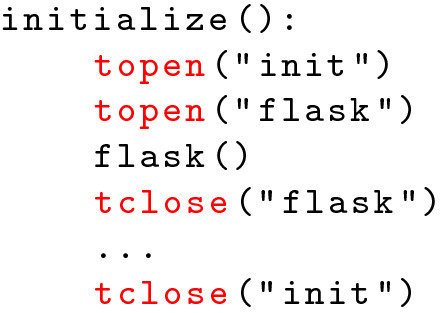
Example of instrumented code

As a first step of refactoring towards a more FaaS-aware implementation, we, therefore, created a simple, lightweight profiling tool that instruments OpenWhisk’s logging facilities and allows to easily include start- and endpoints of relevant functional sections in the code. An accompanying evaluation tool allows to easily analyze respective runtimes^5^. Using these tools, we instrumented the migrated application with a set of tracepoints, see Fig. 2.

**Fig. 3.**
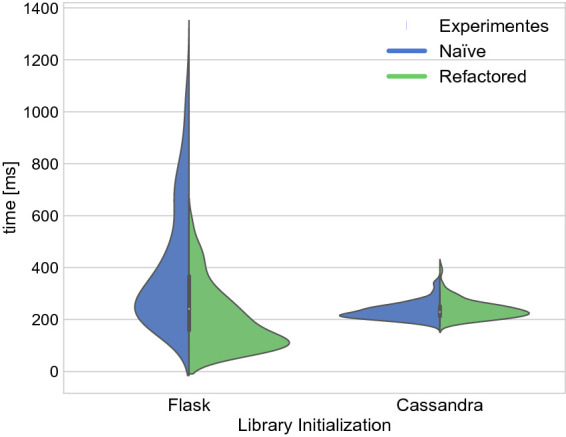
Comparison of cold-start initialization times. “Naïve” refers to the initial migration, see Sect. 3.1. “Refactored” refers to the code-base after the changes described in Sect. 3.3 (Precompute). Results a based on 200 sensor range queries.

We placed each trace-point at potential bottlenecks and points of interest within the code. In particular, we measured object creation, database connection initialization, overall initialization, execution [5], and serialization and de-serialization times. Figure 3 shows exemplary results of these measurements for a simple sensor query, before and after refactoring.

We were quickly able to pin down the root causes of the observed performance degradation based on the gathered information. In particular, we observed that in the initial, naïve approach, the API implementation took substantial time to (re-) initialize certain libraries on every single request. Large portions of these initialization overheads could be attributed to Flask and the Cassandra driver. Accordingly, the inefficient pattern of continuous re-initialization for both Flask and the Cassandra driver was a particular subject of refactoring, described further in the next section.

### Refactoring

The data from the regression detection provided a road-map to address the performance issues in the naïvely migrated application. Based on the identified bottlenecks, the following measures particularly helped us to significantly reduce FaaS-specific overheads:

**Reuse:** We initially focused on reducing the re-initialization of objects and libraries on every single request, see the Reuse tactic in Sect. 4. Specifically, we moved the initialization of most libraries away from the OpenWhisk handler so that initialized libraries remain in memory. We faced some minor challenges as the OpenWhisk runtime did not offer simple mechanisms to execute code before a handler call. However, Python packages allow code execution on imports through including the code to be executed in the __init__ method, which enabled us to shift all expensive initializations to the OpenWhisk runtime creation.

These steps already reduced performance degradations for warm^6^ execution environments significantly.

**Precompute:** Based on a more in-depth analysis of the execution times, we identified that the initialization of both Flask and the Cassandra driver still created notable performance impacts during cold-starts.

For Flask, we managed to precompile most of the Flask object state through the pickle api^7^, which we could store as part of the deployment artifact. Using the precompiled object allows that most of the dynamic computation which Flask performs during server initialization can be removed. The effects of this can be seen in the left part of Fig. 3, see the Precompute tactic in Sect. 4.

We tried to follow a similar strategy for the Cassandra driver. However, we could not reduce the initialization time significantly with this method. Furthermore, we tried to disable thread-pooling and other functionality that is unnecessary in a FaaS context, but these steps also slightly reduced the initialization time.

**Strip:** To address the Cassandra driver’s performance issues, we observed that the Cassandra driver is not used for every request. For instance, an API method only returning sensor metadata based on several query parameters does not need access to the timeseries data stored in Cassandra and could, therefore, be included in a “Cassandra-less” function. Thus, we decided to strip away the Cassandra driver splitting the Flask API into two functions. Of course, this step did not help to reduce the start-up time for Cassandra-related requests but significantly improved the request-response time for all other requests. Significantly decreasing the Cassandra-related overheads for API methods *with* timeseries functionality, however, would have required us to replace or rewrite the Cassandra library. Even though this would have gone beyond the scope of our migration project, this nonetheless illustrates the need for more lightweight, and possibly less feature-rich libraries and drivers in the FaaS context.

## Migration Tactics

Beyond those explicitly mentioned above, we also experimented with further approaches of adjusting our code and the underlying runtime to FaaS-specific givens, which mostly took a similar line, albeit with less significant impacts. Altogether, however, our experiences can be synthesized into five general tactics for FaaSification. Each of these tactics implies different prerequisites, the potential for performance improvements, and development costs.

**Precompute:** This tactic precomputes intermediate results that are included as static content in the deployment package of a function. The tactic requires that the intermediate result does not depend on runtime information and that the size of the intermediate result is comparatively small due to FaaS platform limitations – e.g., 3-250MB for AWS Lambda^8^. In addition, it increases cold-start times with the size of a deployment package [9]. The tactic affects all invocations of a function handler and requires additional development efforts.

**Reuse:** This tactic caches intermediate results over multiple invocations of a function handler on the same function container. It requires that multiple executions occur on the same function container. This tactic is quite simple to implement by storing intermediate results in global class variables of a function handler or on the ephemeral storage available. However, it benefits only a subset of executions, namely those that actually run on the same function container repeatedly. Thus, Reuse becomes less effective with increasing numbers of cold starts. Different approaches for experiment-driven analysis of cold/warm start ratios [3, 5, 9] can indicate the effectiveness of this tactic to developers.

**Strip:** Strip implies that the developer removes source code from the function handler that is initialized but not used on the execution paths of any invocation. It requires that such source code exists and is identifiable by developers. While all invocations benefit, the tactic implies additional development efforts due to profiling and exhaustive testing. We envision that system providers will begin offering specialized lightweight client libraries for short-lived ephemeral compute environments like FaaS platforms.

**Be Lazy:** This tactic is applicable for function handlers with multiple initializations that are not required on all execution paths. The application developer can conditionally initialize by relying on conditional statements in a function handler. As an alternative, a developer can decompose a function into multiple focused functions [1]. The potential for performance improvements depends on the distribution of the different execution paths. It implies additional development efforts and potentially changes the application on an architectural level.

**Replace:** Application developers can resort to re-implementing third-party libraries. A particular form of replacement could be the inclusion of lightweight database connectors within FaaS platforms, allowing FaaS application developers to offload respective functionalities from their code-base. This tactic has no prerequisites and a high potential for improvement but can imply significant development costs. In a FaaS context, the potential additional costs of feature-rich client libraries in terms of higher execution latency and monetary execution costs motivate coexisting lightweight clients with a reduced set of features.

Besides, we argue that a high degree of automation is achievable in order to reduce the development efforts for the different tactics significantly. However, it remains an open question on which level of the technology stack each tactic is applied best. For example, a tactic might be best applied automatically by the cloud platform, integrated with application frameworks, or supported by additional developer tooling in support of FaaSification. We argue that future work should discuss and give guidance on the different associated trade-offs.

## Conclusion

In this paper, we introduced five tactics in support of migrating legacy-applications to FaaS, that we synthesized from a real-world migration effort. We argue that a high degree of automation is achievable in order to reduce the development efforts for migrations in the future. We additionally see that an extension to current FaaS platforms by offloading expensive operations like database connections to the platform could be an area for investigation. Further, we argue that application framework developers could support FaaS best-practices like lazy loading directly. Lastly, we see an opportunity for third-party library vendors to offer more lightweight options aligned with the FaaS context.

## Footnotes

1 https://openwhisk.apache.org/.

2 https://github.com/apache/openwhisk-runtime-python.

3 https://github.com/alexmilowski/flask-openwhisk.

4 Initially we considered more mature frameworks, e.g., Zappa (https://github.com/Miserlou/Zappa). However, OpenWhisk is not supported in most of them.

5 https://bit.ly/2Z4TpsR.

6 Warm execution environments are functions that were deployed by the platform for a previous event. State in these functions can be reused, reducing expensive initializations, that occur during cold-starts [8].

7 https://docs.python.org/3/library/pickle.html.

8 https://docs.aws.amazon.com/lambda/latest/dg/gettingstarted-limits.html.
